# Mechanical Performance and Uniaxial Compressive Behavior of Nano-TiO_2_-Modified Coral Concrete

**DOI:** 10.3390/nano16130824

**Published:** 2026-07-04

**Authors:** Jiahui Wu, Jiakun Zhu, Ao Zhang, Xiaochun Fan

**Affiliations:** 1 School of Civil Engineering and Architecture, Wuhan University of Technology, Wuhan 430070, China; wjh041219@whut.edu.cn (J.W.); a_zhang@whut.edu.cn (A.Z.); fxcfree@126.com (X.F.); 2 The College of Post and Telecommunication, Wuhan Institute of Technology, Wuhan 430070, China

**Keywords:** modified coral concrete, nano-TiO_2_, water environment, mechanical properties, stress–strain relationship

## Abstract

This study investigates the mechanical properties and uniaxial compression behavior of nano-TiO_2_-modified coral concrete (NTCC). Twelve groups of specimens with different nano-TiO_2_ contents were prepared and cured in freshwater, seawater, and oxalic acid environments. Cube compressive strength, splitting tensile strength, and uniaxial compression tests were conducted according to relevant standards. The results indicate that nano-TiO_2_ significantly enhances the mechanical performance of coral concrete. The compressive and tensile strengths initially increased and then decreased with increasing nano-TiO_2_ content, with the maximum strength improvement reaching approximately 22%. Furthermore, increasing the nano-TiO_2_ dosage reduced the brittle failure characteristics of NTCC under compression. The curing environment had a significant influence on the performance of NTCC. Specimens cured in seawater exhibited superior early-age strength, whereas those cured in freshwater achieved the highest later-age strength. The stress–strain response of NTCC under uniaxial compression can be divided into three stages: the elastic stage, elastoplastic stage, and descending stage. Based on the experimental results, an empirical constitutive model was proposed for NTCC. The predicted stress–strain curves showed good agreement with the experimental results, demonstrating the applicability of the proposed model for describing the compressive behavior of NTCC.

## 1. Introduction

With the rapid development of the global economy, the utilization of marine resources has become increasingly important, especially driven by a large number of island construction projects. To cope with high transportation costs and long construction cycles, researchers have explored the feasibility of using local resources such as coral fragments and seawater to prepare concrete [1,2]. This practice not only dramatically reduces construction time but also significantly lowers costs, providing a more economical option for island construction [3,4].

Coral aggregate concrete (CAC) is a concrete prepared using coral aggregate as the aggregate [5]. Several scholars have investigated the basic mechanical properties and durability of coral concrete [6,7,8,9,10,11,12,13], including factors such as compressive strength, tensile strength, stress–strain relationship, and resistance to chloride ion permeability. It has been shown that the shortcomings of coral aggregates, such as high brittleness, high chloride ion content, and poor durability [14,15,16], limit the application of CAC, making the mechanical properties of CAC generally inferior to those of ordinary concrete. To solve this problem, researchers have tried to modify CAC by various means, such as aggregate modification, the use of special cementitious materials to replace ordinary silicate cement, the addition of auxiliary cementitious materials, as well as fibers [16,17,18]. These methods can improve the performance of CAC to different degrees. Among them, the addition of auxiliary cementitious materials and fibers is the most convenient and common, and generally, the auxiliary cementitious materials include fly ash, silica fume, and blast-furnace slag [19].

For example, Zhang et al. [20] concluded that the incorporation of basalt polypropylene fibers reduced the degree of impact damage of coral concrete, especially when these two fibers were mixed. Guo et al. [21] prepared alkali magnesium sulfate cement coral aggregate concrete, whose high toughness of alkali magnesium sulfate cement can effectively improve the brittleness of concrete compared with ordinary cement. Zhang et al. [22] prepared alkali-activated seawater coral aggregate concrete by replacing ordinary silicate cement with alkali-activated materials, which effectively improved its seawater corrosion resistance. In addition, Tanimola et al. [23] investigated nano-modified concrete, where nano-silica accelerates cement hydration and densifies the matrix, while nano-titanium dioxide promotes photocatalytic activity and improves the pore structure, resulting in improved performance and reduced environmental impact. It has been shown that nano-modified concrete has higher durability, flexural and splitting tensile strength, lower water absorption and better pore structure compared to conventional concrete. Similar conclusions were reached by Zhou et al. [24], who used nanosilica to modify recycled aggregate concrete. This suggests that nanotechnology has potential applications in improving the mechanical properties of CAC and provides a new technical way to improve the performance of concrete materials. It is of great research value and engineering significance to investigate the effects of different dosages of nanomaterials on the mechanical properties of CAC.

Most of the above studies currently use seawater or freshwater as the mixing water for CAC. However, the marine environment is complex, and the mixing water used in the preparation of CAC and the external water environment during the subsequent use of the structure may affect the mechanical properties of the concrete. Researchers have also begun to pay attention to the effects of different water environments on the properties of CAC. Fan et al. [25] investigated the variation patterns of mechanical properties of CAC with age under freshwater, seawater and oxalic acid, and the results showed that the compressive strength increased and then stabilized with the increase in curing age in seawater and freshwater, while it increased and then decreased in oxalic acid. Bunyamin et al. [26] investigated the mechanical properties of coral concrete immersed in distilled water and seawater. Chen et al. [27] investigated the compressive strength, mass change and chloride ion concentration at different ages by mixing coral aggregate concrete with seawater and freshwater. The results showed that the early strength of concrete in a seawater environment was higher than that in a freshwater environment, but the stabilized strength was lower than that in a freshwater environment. Da et al. [28] investigated the chloride diffusion behavior of coral concrete in three environments: seawater immersion, wet–dry cycling, and a combination of carbonation and wet–dry cycling. The results showed that carbonation increased chloride diffusion. Qin et al. [29] investigated the healing efficiency of microencapsulated cemented coral sand in freshwater, seawater, and aqueous environments with different pH levels. It was found that microencapsulated cemented coral sand had the highest healing efficiency in freshwater environments, and the healing efficiency was greatly reduced in acidic and alkaline environments. Therefore, it is necessary to study the effect of an acidic water environment on the physical and mechanical properties of coral concrete, in particular, the mechanical properties of coral aggregate concrete in an oxalic acid environment.

In summary, in order to further improve the mechanical properties of CAC in different aqueous environments, this study aims to modify CAC by adding nano-TiO_2_ and investigate the effects of different doping levels of nano-TiO_2_ (0%, 2%, 4% and 6%) on the mechanical properties of modified CAC (NTCC). The study will analyze and evaluate NTCC in freshwater, seawater and oxalic acid environments, and propose the uniaxial compressive stress–strain full curve equations of NTCC based on the existing modified equations of stress–strain relationship, so as to provide a theoretical basis for its application in practical engineering.

## 2. Materials and Methodology

### 2.1. Materials

**Figure 1:**
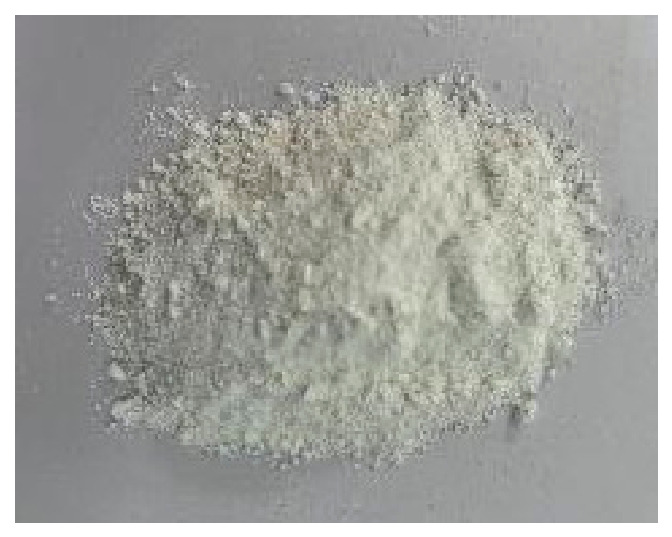
Nano-TiO_2_.

**Figure 2:**
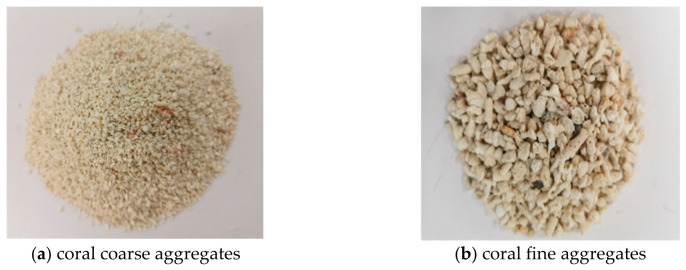
Coral aggregates.

**Figure 3:**
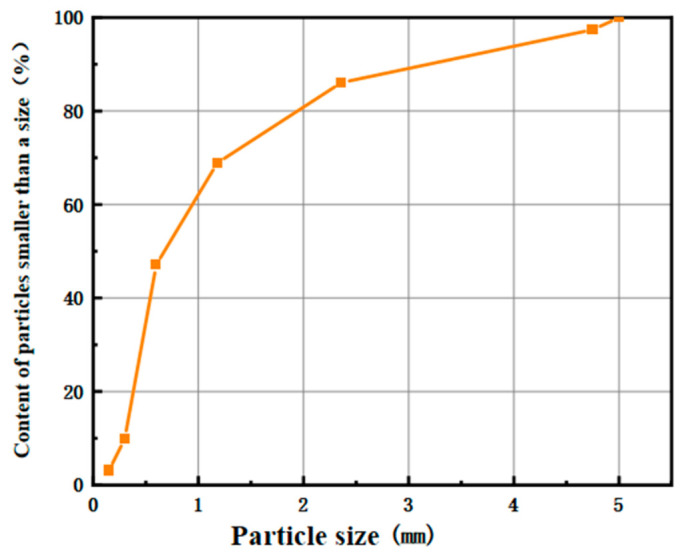
Coral sand particle gradation curve.

The materials employed in this study comprise ordinary silicate cement, fly ash, slag, nano-TiO_2_, coral aggregate, water reducer, artificial seawater, and oxalic acid solution. The ordinary silicate cement (P.O. 52.5) was supplied by Huaxin Cement Co., Ltd. (Huangshi, Hubei, China). Fly ash and slag were obtained from Yangluo Power Plant (Wuhan, China). The water reducer, nano-TiO_2_, oxalic acid, and all chemical reagents used for preparing the artificial seawater were provided by Suzhou Yuante New Materials Co., Ltd. (Suzhou, Jiangsu, China). The primary chemical compositions of the cement, fly ash, and slag are listed in Table 1. The nano-TiO_2_ has an average particle size of 20 nm, a specific surface area of 65 m^2^/g, and a packing density of 0.24 g/cm^3^, as illustrated in Figure 1. The coral aggregate was procured from the South China Sea, as illustrated in Figure 2. The fine aggregate was a continuously graded coral sand with a particle size of 0.15–4.75 mm, exhibiting a fineness modulus of 2.87, Zone II gradation, and medium sand. The gradation curve is depicted in Figure 3. The coarse coral aggregate was selected to have a particle size between 4.75 and 8 mm. The water-reducing agent is a polycarboxylic acid of high performance, with a water-reducing rate in excess of 25%.

The freshwater is tap water from Wuhan City, and the artificial seawater is formulated with reference to the composition of seawater in the South China Sea. The composition of the artificial seawater is presented in Table 2. An oxalic acid solution was prepared by combining artificial seawater and an oxalic acid reagent to create a 0.1 mol/L oxalic acid solution.

### 2.2. Mix Proportions and Specimen Preparation

This paper presents the results of an investigation into the effects of different water environments (freshwater, seawater, and oxalic acid environments) and nano-TiO_2_ dosage (i.e., 0%, 2%, 4%, and 6%) on the mechanical and uniaxial compression properties of modified coral concrete. The investigation was conducted using the loose volume design method, with the objective of guaranteeing the working properties of the concrete. A total of 12 groups of coral concrete specimens with varying ratios were designed, and the corresponding fits are presented in Table 3. In the numbering system, NTCC represents nano-TiO_2-_modified coral concrete. The letters F, S, and O indicate the preparation and curing of the test environments: freshwater, seawater, and oxalic acid solution, respectively. The numbers correspond to the mass fraction of nano-TiO_2_ in each specimen. In the case of the freshwater environment group, the specimen blocks were prepared and cured in freshwater. In the case of the seawater environment group, the specimen blocks were prepared and cured in seawater. The test blocks were prepared and cured in an oxalic acid solution with a concentration of 0.1 mol/L for the oxalic acid environment group. The test blocks were prepared and cured in a solution of 0.1 mol/L oxalic acid. For example, NTCC-F2 represents a modified coral concrete with a 2% mass fraction of nano-TiO_2_, prepared and cured using freshwater.

Given the low density of nano-TiO_2_, it is susceptible to being dispersed by air flow. Therefore, it is essential to conduct the dispersion of nano-TiO_2_ in an environment free from wind. Additionally, due to their extremely fine particle size, they cannot be evenly distributed by dry mixing in different concrete mixers or even mortar mixers. This results in the nano-TiO_2_ “sticking to the wall,” which reduces the final concentration of nano-TiO_2_ in the cementitious material. This incomplete dispersion may impair the full functionality of nano-TiO_2_. Accordingly, the dispersion method of nano-TiO_2_ in this test is as follows: First, the requisite quantities of water and water-reducing agent are weighed and combined. This mixture is then agitated for one minute. Subsequently, the nano-TiO_2_ is incorporated into the aforementioned mixture, which is then agitated for three minutes to ensure thorough dissolution of the nano-TiO_2_. This process yields the nano-TiO_2_ suspension. The preparation process of coral concrete is as follows: First, the raw materials are weighed according to the ratio of each group using a forced mixer. Then, a mixed suspension of nano-TiO_2_, water-reducing agent, and mixing water is prepared. And, the coral coarse aggregate, coral sand, and 1/2 parts of the mixed suspension are introduced into the mixer and mixed for 3 min. Upon completion of the mixing process, the remaining mixed suspension, cement, fly ash, and slag are added and mixed for an additional 3 min. Prior to pouring the concrete, a layer of mold release oil was applied to the inner surface of the mold. The finished mixed concrete was then poured into the prepared mold and finally vibrated into shape on the vibrating table with a vibration time of 30 s. Following a 24 h curing period under standard conditions, the specimens were removed from the molds and subjected to immersion curing. The specimens subjected to the freshwater mixture were placed in freshwater immersion curing, while those subjected to the seawater mixture were placed in seawater immersion curing. While those treated with oxalic acid were soaked in oxalic acid for 7 days and 28 days, after which they were removed to undergo the subsequent tests.

In this study, 12 groups of cubic specimens with dimensions of 100 mm × 100 mm × 100 mm and prismatic specimens of 100 mm × 100 mm × 300 mm were prepared. A total of nine cubic specimens, each measuring 100 mm × 100 mm × 100 mm, were prepared for testing. Of these, six were subjected to 7-day and 28-day cubic compressive strength tests, respectively, while the remaining three were subjected to split tensile strength tests. Three prismatic specimens, each measuring 100 mm × 100 mm × 300 mm, were subjected to a uniaxial compression test.

### 2.3. Test Procedures

#### 2.3.1. Cube Compressive and Splitting Tensile Tests

**Figure 4:**
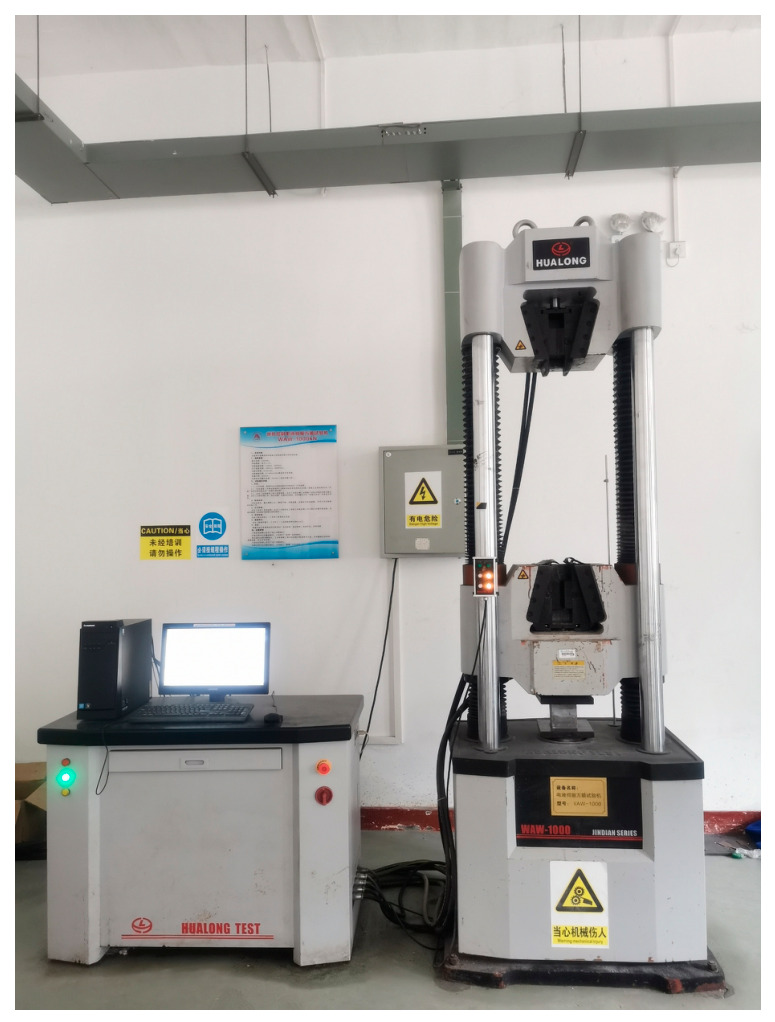
Cube compressive strength test.

**Figure 5:**
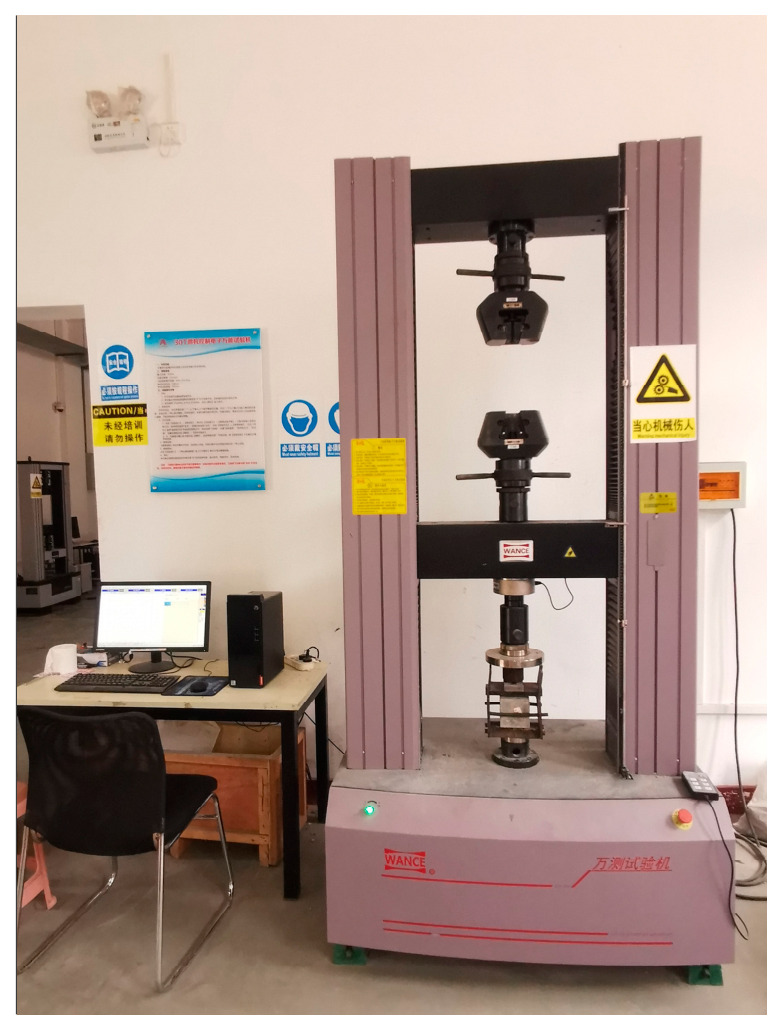
Splitting tensile strength test.

The test is conducted in accordance with the Standard for Test Methods of Physical and Mechanical Properties of Ordinary Concrete (GB/T50081-2019 [30]). The cubic compressive strength test is conducted using a 100 T hydraulic servo universal testing machine, as illustrated in Figure 4. The test employs a continuous and uniform loading method, with a loading rate of 8 kN/s. The splitting tensile strength test is conducted using a 30T hydraulic servo universal testing machine, as illustrated in Figure 5. The surface of the upper and lower bearing plates of the press and the concrete specimen are initially wiped with a rag, and a flatter concrete surface is selected as the splitting surface. The center line was then marked with a marker on the aforementioned surface. The concrete specimen was placed in the center of the fixture, with the fixture’s upper and lower arc-shaped pads and the concrete specimen’s splitting surface marking aligned. Subsequently, a 2 mm thick shim was placed between the circular shim and the concrete sample. Finally, the placement of the concrete specimen was confirmed to ensure that the centerline of the split surface marking was aligned with the centerline of the arc-shaped pads and spacers. The test was conducted using a continuous and uniform loading method, with a loading rate of 1.5 kN/s.

#### 2.3.2. Uniaxial Compression Test

Prior to the commencement of the testing process, each specimen is subjected to a thorough examination. The elevated portions on the edge of the compressed end surface are subjected to grinding with a grinder, with the objective of ensuring that the compressed surface is flat and smooth. It is imperative to ascertain whether the surface of the test piece exhibits any holes or cracks. In the event of the presence of a substantial aperture, it is necessary to seal it with epoxy resin. In order to prepare the rough surface of the test piece for bonding, the raised coarse aggregate must first be roughly ground with a grinder. This is followed by a second grinding stage with an abrasive belt machine, which ensures that the position of the bonded strain gauge is smooth and flat. It is recommended that the strain gauges be bonded in the center of the four sides of the test piece, in both the horizontal and vertical directions. It is of the utmost importance to ensure that no air bubbles remain at the bonding surface of the strain gauge and the test piece during the bonding process. Following the bonding process, it is advisable to utilize a multimeter to ascertain whether the strain gauges have developed short circuits or open circuits.

**Figure 6:**
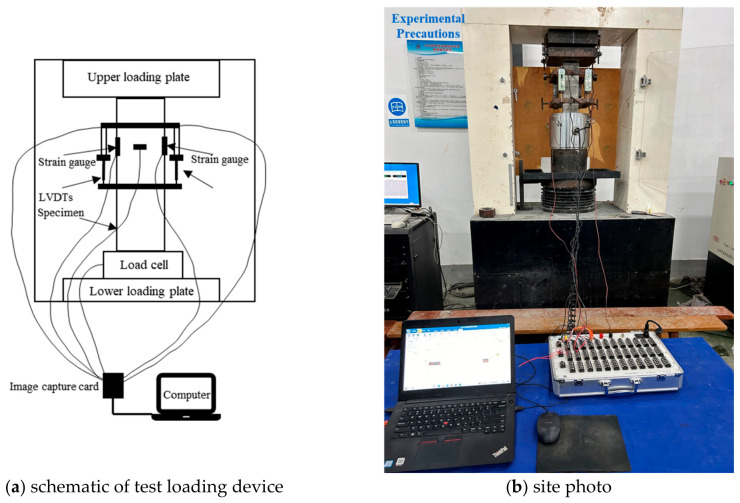
Uniaxial compression test loading device.

In this test, the displacement control mode with a rate of 0.002 mm/s is selected for analysis. A 300T hydraulic servo universal testing machine is employed for the test, with a load cell, displacement transducer, and collector. The load cell utilizes a load cell, the displacement transducer employs the YHD-50 type, and the collector utilizes the Donghua static strain test system to collect signals from the aforementioned components. In the initial stage of the test, the load cell is positioned at the center of the universal testing machine. The displacement sensor is then secured with two steel hoops, which are subsequently fixed at a distance of one-third from each other on the upper and lower surfaces of the specimen. The specimen is then placed in the center of the load cell, and the strain gauges are welded to the collector connecting line. As illustrated in Figure 6, the specimen is prepared for balancing processing by modulating the collector.

#### 2.3.3. Mercury Intrusion Porosimetry (MIP) Analysis

To further investigate the pore structure characteristics of nano-TiO_2_-modified coral concrete, mercury intrusion porosimetry (MIP) was employed. Dried concrete specimens were carefully crushed, and representative fragments with volumes ranging from 0.5 to 1.0 cm^3^ were selected for testing. The pore size distribution, critical pore diameter, and total porosity of the samples were determined using an AutoPore V 9600 mercury intrusion porosimeter (Micromeritics Instrument Corporation, Norcross, GA, USA). Prior to measurement, the specimens were placed in a dilatometer, accurately weighed, and then subjected to mercury intrusion under controlled pressures. The operating pressure ranged from 0.1 psia to 33,000 psia (approximately 0.0007 MPa to 228 MPa). The surface tension and contact angle of mercury were set at 0.485 N/m and 130°, respectively.

## 3. Results and Discussions

### 3.1. Cubic Compression Strength

#### 3.1.1. Effect of Nano-TiO_2_ Content

**Figure 7:**
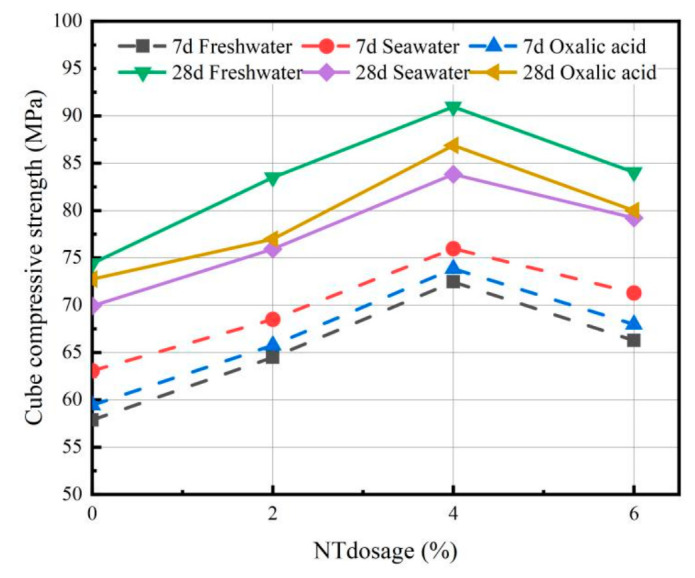
Cubic compressive strength of modified coral concrete versus nano-TiO_2_ dosage.

Figure 7 illustrates the variation in cube compressive strength of modified coral concrete at 7 and 28 days with nano-TiO_2_ content in different water environments. As can be observed from the figure, an increase in nano-TiO_2_ content is associated with a trend of initial increase and subsequent decrease in cube compressive strength of NTCC at 7 and 28 days in different water environments. At day 7, in freshwater, the compressive strength of NTCC cubes with nano-TiO_2_ dosages of 2%, 4% and 6% was found to be 11.45%, 25.22% and 14.53% higher, respectively, than that of the control. In seawater, the compressive strength of NTCC cubes with 2%, 4% and 6% nano-TiO_2_ increased by 8.63%, 20.47% and 13.03%, respectively, in comparison to the strength of cubes with 0% nano-TiO_2_. In an oxalic acid environment, the compressive strength of NTCC cubes with nano-TiO_2_ dosages of 2%, 4% and 6% exhibited an increase of 10.63%, 24.25% and 14.36%, respectively, in comparison to the 0% dosage. At 28 days, the compressive strength of NTCC cubes doped with 2%, 4% and 6% nano-TiO_2_ was observed to be 12.21%, 22.23% and 12.94% higher than that of NTCC-F0 cubes, respectively. In seawater, the compressive strength of NTCC cubes doped with 2%, 4% and 6% nano-TiO_2_ is observed to be 8.61%, 19.89% and 13.29% higher than that of NTCC-S0 cubes, respectively. In an oxalic acid environment, the compressive strength of NTCC cubes doped with nano-TiO_2_ at 2%, 4% and 6% increased by 5.81%, 19.43% and 9.96%, respectively, in comparison to the compressive strength of NTCC-O0 cubes. The compressive strength of the nanometer-sized TiO_2_ doped with 4% reached its maximum value at 7 days and 28 days in freshwater, seawater and oxalic acid environments.

#### 3.1.2. Effect of Water Environment

**Figure 8:**
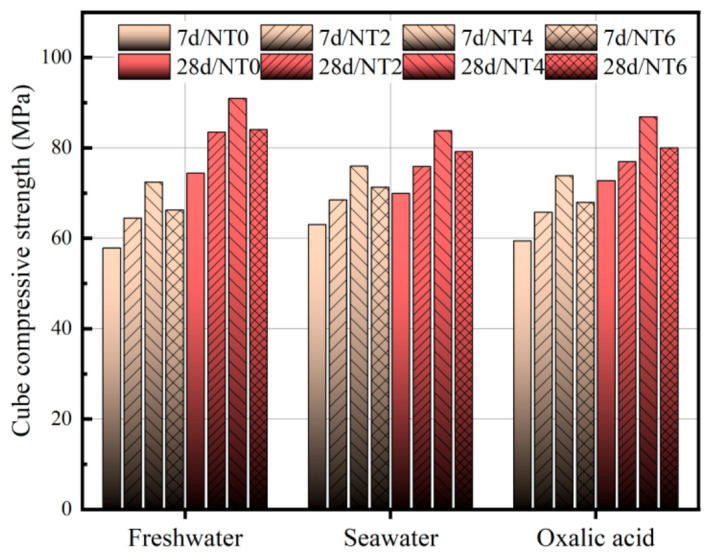
Cubic compressive strength of modified coral concrete as a function of aqueous environment.

Figure 8 illustrates the compressive strength of modified coral concrete cubes in diverse aqueous environments. As illustrated in the figure, when the curing age is 7d, the compressive strength of NTCC cubes with varying nano-TiO_2_ dosages in a seawater environment is superior to that of NTCC cubes with corresponding nano-TiO_2_ dosages in oxalic acid and freshwater environments. However, at the 28-day curing age, the compressive strength of NTCC cubes with different dosages of nano-TiO_2_ was highest in the freshwater environment, followed by the oxalic acid environment, and lowest in the seawater environment. At 7 days, the compressive strength of the NTCC cubes in the seawater environment with nano-TiO_2_ dosages of 0%, 2%, 4%, and 6% was observed to be 9.00%, 6.23%, 4.86%, and 7.57% higher than that in the freshwater environment, respectively. Additionally, the compressive strength in the seawater environment with nano-TiO_2_ dosages of 0%, 2%, 4%, and 6% was found to be 6.11%, 4.18%, 2.88%, and 4.88% higher than that in the oxalic acid environment, respectively. At 28 days, the NTCC cube compressive strength in seawater was observed to be 6.03%, 9.05%, 7.83%, and 5.75% lower than in freshwater for nano-TiO_2_ dosages of 0%, 2%, 4%, and 6%, respectively. Similarly, the NTCC cube compressive strength in oxalic acid was found to be 2.22%, 7.80%, 4.47%, and 4.81% lower than in freshwater for nano-TiO_2_ dosages of 0%, 2%, 4%, and 6%, respectively. Furthermore, the compressive strength of NTCC cubes in freshwater at 7 days can reach 77.77%, 77.24%, 79.67%, and 78.86% of the compressive strength of NTCC cubes in freshwater at 7 days when the dosage of nano-TiO_2_ is 0%, 2%, 4%, and 6%, respectively. The compressive strength of NTCC cubes in seawater at 7 days can reach 90.20%, 90.22%, 90.64% and 90.00% of the compressive strength of 28-day concrete cubes, respectively. The compressive strength of NTCC cubes in oxalic acid at 7 days can reach 81.70%, 85.42%, 85.00%, and 84.97% of the compressive strength of 28-day concrete cubes, respectively. In comparison to freshwater and oxalic acid environments, the seawater environment demonstrates a higher early compressive strength at 7 days.

### 3.2. Split Tensile Strength

#### 3.2.1. Effect of Nano-TiO_2_ Content

**Figure 9:**
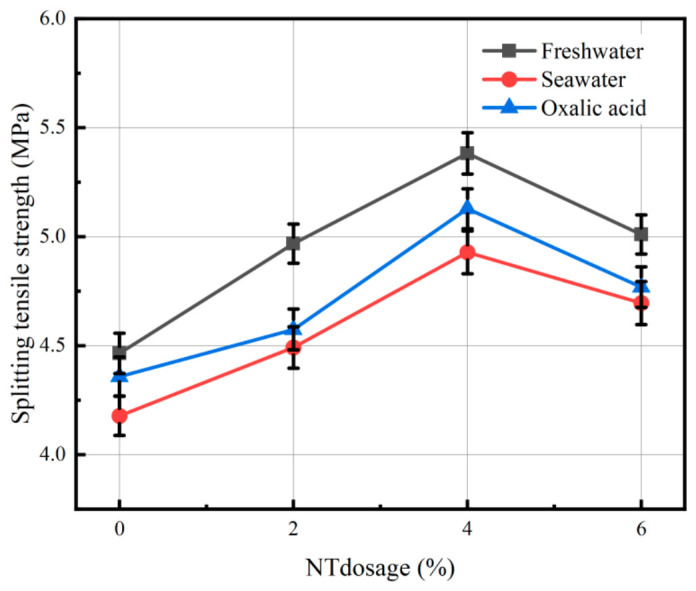
Splitting tensile strength of modified coral concrete versus nano-TiO_2_ dosage.

Figure 9 illustrates the impact of nano-TiO_2_ dosage on the splitting tensile strength of modified coral concrete. As illustrated in the figure, the splitting tensile strength of NTCC in diverse aqueous environments exhibits a dual trend: initially increasing with nano-TiO_2_ dosage, and subsequently declining. At a dosage of 4%, the splitting tensile strength of modified coral concrete in various water environments attains its maximum value, which is in accordance with the variation law of cube compressive strength with the dosage of nano-TiO_2_. Furthermore, the maximum splitting tensile strength of modified coral concrete in freshwater, seawater, and oxalic acid environments is 20.55%, 17.96%, and 17.67% higher, respectively, than its minimum value in the corresponding water environment. In freshwater environments, the addition of nano-TiO_2_ at dosages of 2%, 4%, and 6% resulted in a notable enhancement in the splitting tensile strength of the modified coral concrete, with increases of 11.27%, 20.55%, and 12.21%, respectively, compared to the modified coral concrete with a 0% nano-TiO_2_ dosage. In seawater environments, the application of nano-TiO_2_ at dosages of 2%, 4%, and 6% resulted in increases of 7.51%, 17.96%, and 12.39%, respectively, in the splitting tensile strength of the modified coral concrete. The modified coral concrete exhibited an increase of 4.96%, 17.67%, and 9.42%, respectively, when the dosage of nano-TiO_2_ was 2%, 4%, and 6% in the oxalic acid environment. However, when the dosage of nano-TiO_2_ is 6%, the splitting tensile strength of the modified coral concrete in freshwater, seawater, and oxalic acid environments is observed to be 6.92%, 4.73%, and 7.01% lower, respectively, than that of the control sample.

#### 3.2.2. Effect of Water Environment

**Figure 10:**
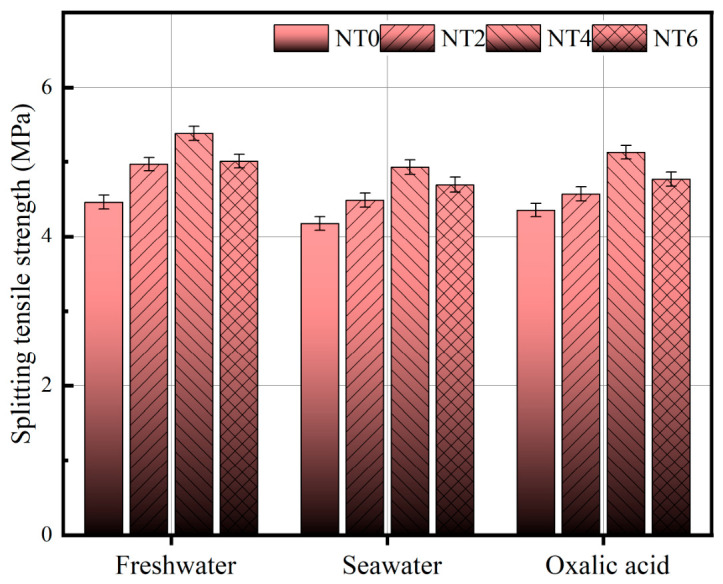
Splitting tensile strength of modified coral concrete versus the water environment.

Figure 10 illustrates the split tensile strength of the modified coral concrete in various aqueous environments. As illustrated in the figure, the split tensile strength of the modified coral concrete is highest in freshwater, second in oxalic acid, and lowest in seawater when the same dosage of nano-TiO_2_ is applied. Furthermore, the mean values of the splitting tensile strength of the modified coral concrete under the four dosages of nano-TiO_2_ are highest in the freshwater group, followed by the oxalic acid group and the seawater group. The splitting tensile strength of the modified coral concrete in a seawater environment is 6.42%, 9.59%, 8.43%, and 6.28% lower than that in a freshwater environment when the dosage of nano-TiO_2_ is 0%, 2%, 4%, and 6%, respectively. In the oxalic acid environment, the splitting tensile strength of modified coral concrete is 2.40%, 7.92%, 4.72%, and 4.82% lower than that of modified coral concrete in freshwater, respectively, when the dosage of nano-TiO_2_ is 0%, 2%, 4%, and 6%, respectively.

### 3.3. Uniaxial Compression Failure Patterns

**Figure 11:**
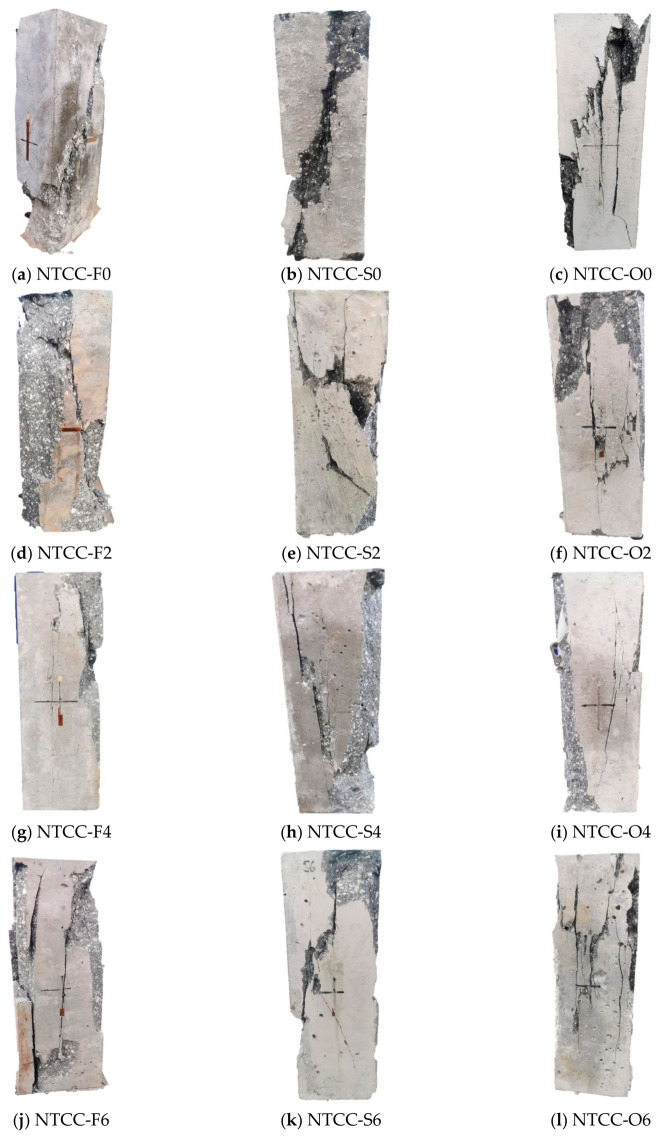
NTCC axial compression damage patterns.

Figure 11 illustrates the axial compression failure modes of various NTCC types. At the outset of the loading procedure, a multitude of minute fissures manifested in the vicinity of the diagonals of the test block. As the load increased gradually, the cracks continued to propagate to the ends of the diagonals, accompanied by a notable increase in the number, length, and width of the cracks. Concurrently, a multitude of minute fissures manifested in the axial region of the test block. As the loading continued, the cracks continued to lengthen and widen, ultimately forming a large crack through the diagonal, resulting in the failure of the test block. As illustrated in the figure, all test pieces were destroyed diagonally, with some of the blocks peeling off. As with the compression failure of the cube, the degree of damage to the test piece initially decreased and then increased with the increase in the nano-TiO_2_ dosage under the same water environment. Additionally, the width and number of cracks exhibited a gradual decrease and then an increase, respectively. The lowest degree of damage and narrowest cracks were observed at a dosage of 4%. The greatest degree of damage was observed in the seawater environment under the same nano-dosage, followed by oxalic acid, and the lowest degree of damage was observed in freshwater.

### 3.4. Uniaxial Compression Stress–Strain Curve

**Figure 12:**
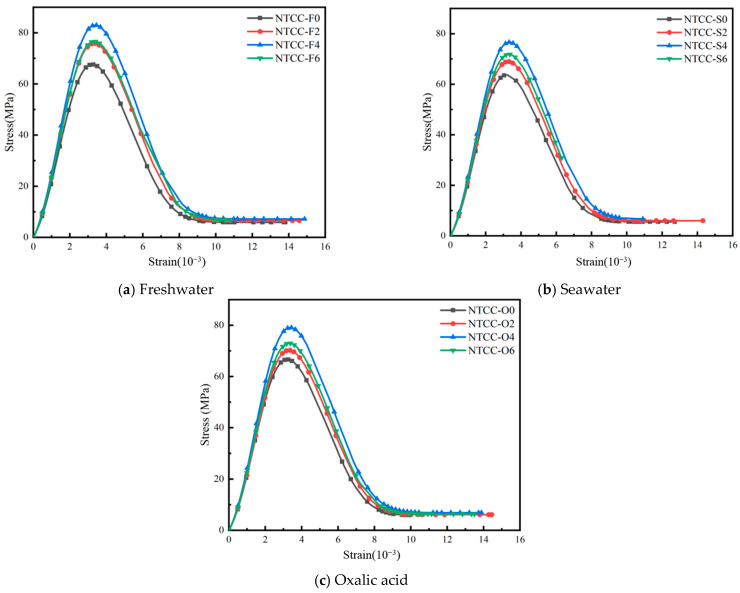
Uniaxial compression stress–strain curve of NTCC with different nano-TiO_2_ doping levels.

Figure 12 shows the complete stress–strain curves of NTCC under uniaxial compression for different nano-TiO_2_ dosages and curing environments. Overall, these curves can be divided into three distinct stages. In the initial phase, spanning the initial loading phase to approximately 90% of the peak stress, the curve exhibits a linear increase. In the second stage, as the load increases gradually, the rate of growth of the curve diminishes. Subsequently, following the attainment of the peak load, a downward trajectory is initiated. This is due to the fact that during the initial phase of loading, concrete displays approximately linear elastic properties, with a direct proportional relationship between stress and strain. As the external load increases, the stress–strain relationship deviates from linearity and enters the inelastic stage, at which point strain is divided into elastic and plastic components. The formation and propagation of microcracks within the concrete matrix are initiated, particularly in regions exhibiting microscopic material imperfections, such as pores. Further increases in load result in additional plastic deformation, accompanied by the continuous expansion and interconnection of microcracks, and a notable decrease in the slope of the stress–strain curve. Once the peak stress is reached, the stress–strain curve enters a downward phase, accompanied by a decrease in the bearing capacity of the concrete. While the stress level declines, the strain continues to increase, resulting in a more pronounced downward slope on the stress–strain curve. Concurrently, macroscopic cracks emerge on the surface of the concrete and gradually expand.

Figure 12 also compares the influence of nano-TiO_2_ dosage and curing environment on the shape of the stress–strain curves. As illustrated in the figure, the ascending order of the slope of the first stage of the curve in the three water environments is NTCC, NTCC-F6, NTCC-F2, and NTCC-F0. This is due to the fact that the concrete displays excellent elasticity during the initial phase of the curve, and the slope is positively correlated with the elastic modulus. Consequently, this is in accordance with the law governing the change in elastic modulus. As the stress–strain continues to increase and reaches the peak stress, the specimen will undergo a brittle failure, resulting in a sharp decline in the curve. The rate of decrease is as follows: 0% nano-TiO_2_ dosage, 2% nano-TiO_2_ dosage, 6% nano-TiO_2_ dosage, and 4% nano-TiO_2_ dosage. This is due to the fact that, subsequent to the specimen’s failure, the lower the ductility of the specimen, the steeper the slope of the curve. The full stress–strain curves of NTCC under uniaxial compression in different water environments demonstrate that the slope of the ascending section of the curve is freshwater > oxalic acid > seawater, which is in accordance with the law of change in the elastic modulus of the test piece in different water environments. Given that the test piece exhibited the least ductility in seawater, followed by oxalic acid, and the greatest in freshwater, the rate of decline in seawater was the most pronounced in the descending section of the curve.

### 3.5. Peak Stress and Peak Strain

#### 3.5.1. Effect of Nano-TiO_2_ Content

**Figure 13:**
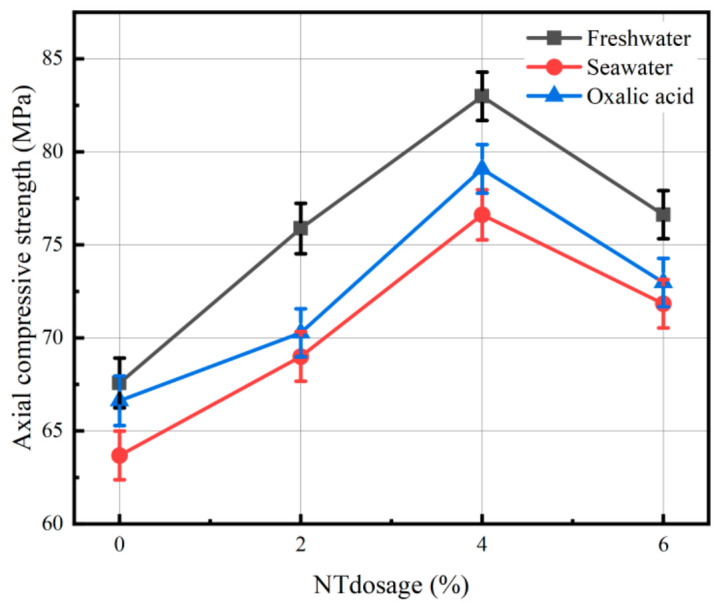
Relationship between the compressive strength of modified coral concrete and the dosage of nano-TiO_2_.

Figure 13 illustrates the impact of nano-TiO_2_ dosage on the axial compressive strength of modified coral concrete. As illustrated in the figure, an increase in the dosage of nano-TiO_2_ is observed to result in an initial rise in the axial compressive strength of the NTCC, followed by a subsequent decline. At a dosage of 4%, the axial compressive strength of the NTCC reaches a maximum. In freshwater environments, the addition of nano-TiO_2_ at dosages of 2%, 4%, and 6% has been observed to enhance the axial compressive strength of concrete by 12.29%, 22.80%, and 13.39%, respectively, in comparison to the strength of concrete without nano-TiO_2_ supplementation. In a seawater environment, the concrete’s axial compressive strength exhibits an increase of 8.35%, 20.32%, and 12.81%, respectively, when the dosage of nano-TiO_2_ is 2%, 4%, and 6%, in comparison to the control group with no nano-TiO_2_ addition. In an oxalic acid environment, the addition of 2%, 4%, and 6% nano-TiO_2_ has been observed to result in an increase in the concrete’s axial compressive strength, with values of 5.48%, 18.70%, and 9.55%, respectively, when compared to the control sample with no nano-TiO_2_. When the dosage of nano-TiO_2_ is 6%, the concrete’s axial compressive strength in freshwater, seawater, and oxalic acid environments is observed to decrease by 7.66%, 6.25%, and 7.71%, respectively, in comparison to the dosage of 4%.

**Figure 14:**
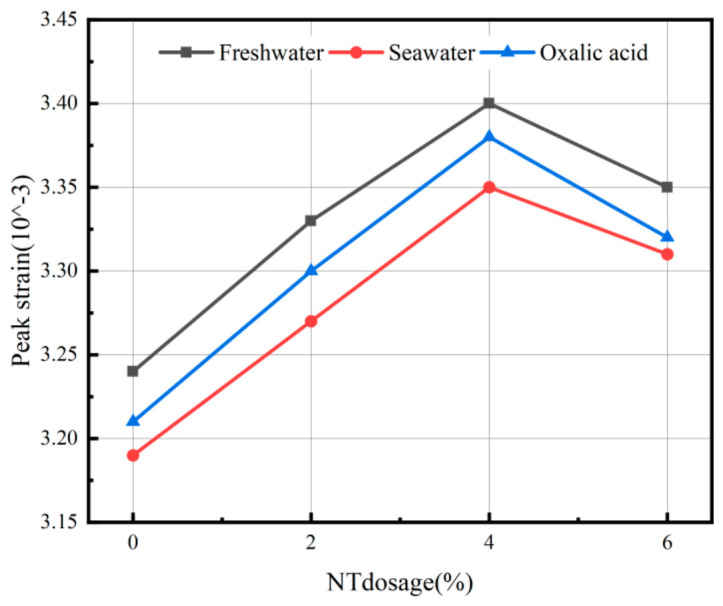
Relationship between peak strain of modified coral concrete and dosage of nano-TiO_2_.

Figure 14 illustrates the relationship between the peak strain of modified coral concrete and the dosage of nano-TiO_2_. As illustrated in the figure, the peak strain of modified coral concrete in diverse water environments exhibits a pattern of initial increase and subsequent decline with the augmentation of nano-TiO_2_ dosage. The peak strain of modified coral concrete attains its maximum value when the dosage reaches 4%. In freshwater, the peak strain of the concrete is increased by 2.78%, 4.94%, and 3.40%, respectively, when the dosage of nano-TiO_2_ is 2%, 4%, and 6% compared to when the dosage of nano-TiO_2_ is 0%. In seawater, the peak strain of the concrete is increased by 2.51%, 5.02%, and 3.76%, respectively, when the dosage of nano-TiO_2_ is 2%, 4%, and 6% compared to when the dosage of nano-TiO_2_ is 0%. In the oxalic acid environment, the peak strain of the concrete is observed to be 2.80%, 5.30%, and 3.43% higher than that of the nano-TiO_2_ dosage of 0% when the dosage of nano-TiO_2_ is 2%, 4%, and 6%, respectively. When the dosage of nano-TiO_2_ is 6%, the peak strain of the concrete in freshwater, seawater, and oxalic acid environments is 1.47%, 1.19%, and 1.78% lower, respectively, than that of the dosage of 4%.

#### 3.5.2. Effect of Water Environment

**Figure 15:**
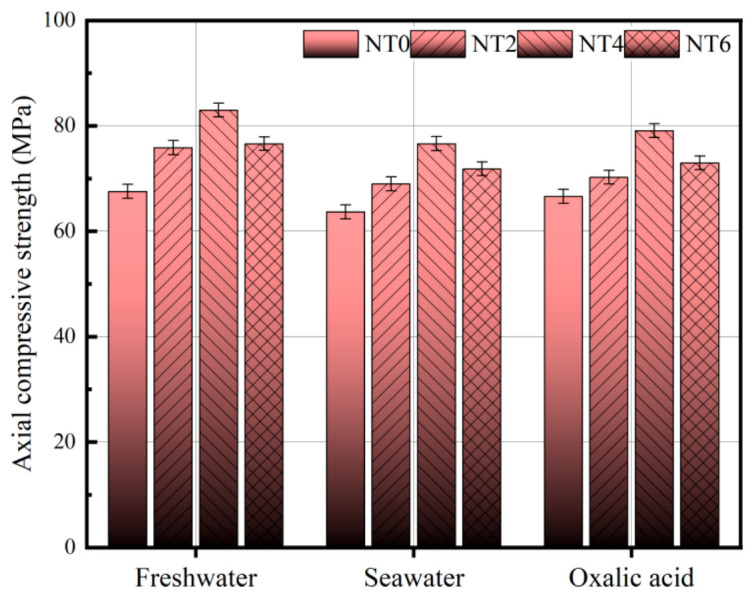
Relationship between the compressive strength of modified coral concrete and the water environment.

Figure 15 illustrates the axial compressive strength of the modified coral concrete in various aqueous environments. As illustrated in the figure, the axial compressive strength of the modified coral concrete with an identical dosage of nano-TiO_2_ is greatest in a freshwater environment, second in an oxalic acid environment, and lowest in a seawater environment. Furthermore, the mean values of axial compressive strength of the modified coral concrete with the four nano-TiO_2_ dosages, in descending order, were as follows: freshwater, oxalic acid and seawater groups, respectively. The compressive strength of the modified coral concrete in the seawater environment was observed to be 5.76%, 9.07%, 7.66%, and 6.25% lower than that in the freshwater environment, respectively, when the nano-TiO_2_ dosages were 0%, 2%, 4%, and 6%. When the nano-TiO_2_ dosages were 0%, 2%, 4%, and 6%, the compressive strength of the coral concrete in the oxalic acid environment was found to be 1.41%, 7.39%, 4.70%, and 4.75% lower, respectively, than that in the freshwater environment. In comparison to freshwater, the greatest reduction in axial compressive strength of coral concrete under seawater and oxalic acid can reach 9.07% and 7.39%, respectively. The maximum reduction occurs at 2% nano-TiO_2_ doping. The underlying microscopic mechanisms responsible for these differences are discussed in detail in Section 3.9.

**Figure 16:**
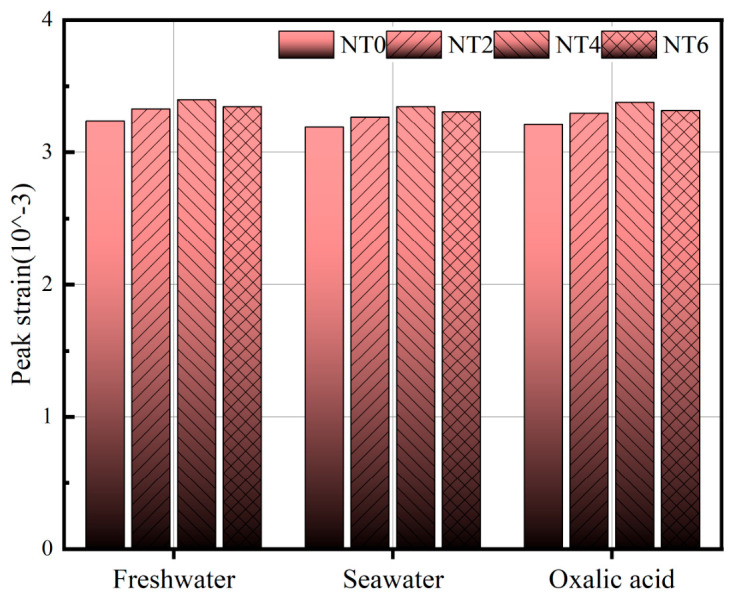
Relationship between peak strain of modified coral concrete and water environment.

Figure 16 illustrates the peak strains of the modified coral concrete in various aqueous environments. As illustrated in the figure, the peak strain of the modified coral concrete with an identical dosage of nano-TiO_2_ is the most pronounced in a freshwater environment, followed by an oxalic acid environment and then a seawater environment. Furthermore, the mean peak strains of the modified coral concrete under the four nano-TiO_2_ dosages, in descending order, are the freshwater group, the oxalic acid group, and the seawater group. When the nano-TiO_2_ dosages were 0%, 2%, 4%, and 6%, the peak strain in the freshwater environment was 1.54%, 1.80%, 1.47%, and 1.19% higher than that in the seawater environment, respectively. Similarly, the peak strain in the freshwater environment was 0.93%, 0.90%, 0.59%, and 0.90% higher than that in the oxalic acid environment, respectively. This pattern of change is consistent with the pattern of change observed in the basic mechanical strength. The primary reason for this phenomenon is that the interaction between Cl^−^, ${SO}_{4}^{2-}$, ${C_{2}O}_{4}^{2-}$, Mg^2+^ and H^+^ in the seawater and oxalic acid environment result in a change to the internal microstructure of the coral concrete, which in turn affects the ductility of the NTCC.

### 3.6. Elastic Modulus

#### 3.6.1. Effect of Nano-TiO_2_ Content

**Figure 17:**
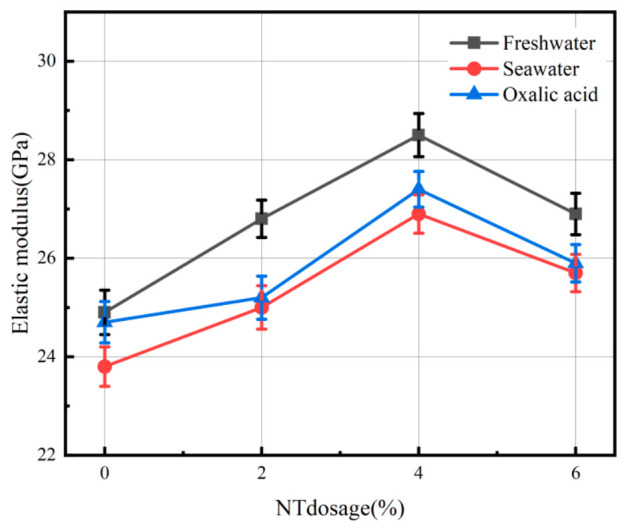
Relationship between the elastic modulus of modified coral concrete and the amount of nano-TiO_2_ added.

Figure 17 illustrates the correlation between the elastic modulus of modified coral concrete and the dosage of nano-TiO_2_. As illustrated in the figure, the elastic modulus of modified coral concrete in diverse water environments exhibits a pattern of initial increase followed by a decline with rising nano-TiO_2_ dosage. The elastic modulus of modified coral concrete attains its maximum value at a dosage of 4%. This change law is consistent with the change laws of compressive strength, tensile strength, and flexural strength. In a freshwater environment, the modulus of elasticity of concrete exhibited a notable increase of 7.63%, 14.46% and 8.03% at 2%, 4% and 6% nano-TiO_2_ dosage in a freshwater environment, respectively, in comparison to the nano-TiO_2_ dosage of zero; in a seawater environment, the elastic modulus of the concrete is observed to be 5.04%, 13.03%, and 7.98% higher than the control when the nano-TiO_2_ content is 2%, 4%, and 6%, respectively; in the oxalic acid environment, the elastic modulus of the concrete was 2.02%, 10.93% and 4.86% higher than the control when the nano-TiO_2_ dosage was 2%, 4% and 6%. The elastic modulus of concrete in freshwater, seawater, and oxalic acid environments is 5.61%, 4.46%, and 5.47% lower, respectively, when the nano-TiO_2_ content is 6% compared to a nano-TiO_2_ content of 4%. Therefore, an increase in the dosage of nano-TiO_2_ results in a gradual reduction in porosity within the coral concrete structure. However, an excess of nano-TiO_2_ agglomerates leads to a decline in the pore-filling effect. Consequently, the compressive strength of the modified coral concrete initially increases and subsequently declines with an elevated nano-TiO_2_ dosage. Similarly, the elastic modulus of the modified coral concrete also initially increases and subsequently declines with an elevated nano-TiO_2_ dosage.

#### 3.6.2. Effect of Water Environment

**Figure 18:**
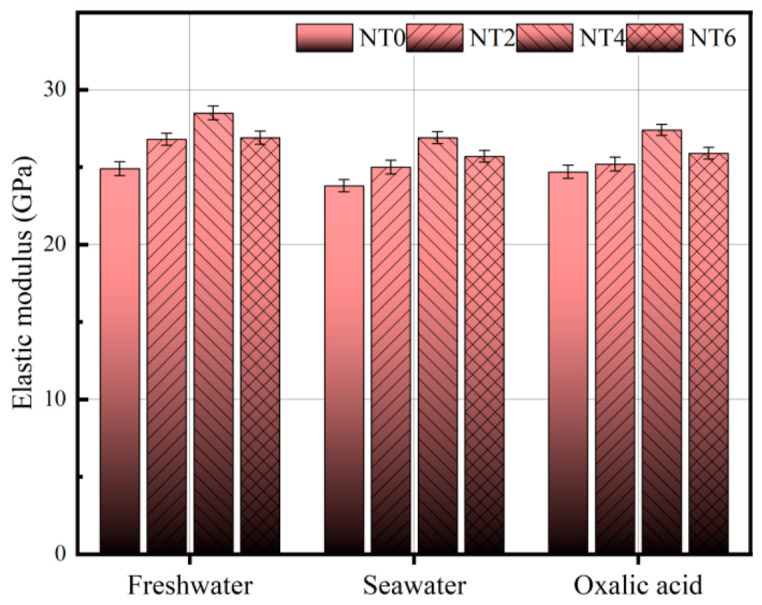
Relationship between the elastic modulus of modified coral concrete and the water environment.

Figure 18 illustrates the elastic modulus of modified coral concrete in various aqueous environments. As illustrated in the figure, the elastic modulus of modified coral concrete with an identical dosage of nano-TiO_2_ is greatest in freshwater, followed by oxalic acid, and lowest in seawater. Furthermore, the mean elastic modulus of modified coral concrete with four nano-TiO_2_ dosages, from highest to lowest, is observed in the freshwater group, oxalic acid group, and seawater group, respectively. When the nano-TiO_2_ content is 0%, 2%, 4%, and 6%, the elastic modulus of the modified coral concrete in the seawater environment is observed to be 4.42%, 6.72%, 5.61%, and 4.46% lower than that in the freshwater environment, respectively. Similarly, the elastic modulus of the coral concrete in the oxalic acid environment is found to be 0.80%, 5.97%, 3.86%, and 3.72% lower than that in the freshwater environment, respectively. In comparison to freshwater, the maximum reduction in the modulus of elasticity of coral concrete in the presence of seawater and oxalic acid can reach 6.72% and 5.97%, respectively. These maximum decreases occurred at a nano-TiO_2_ dosage of 2%.

### 3.7. Poisson Ratio

#### 3.7.1. Effect of Nano-TiO_2_ Content

**Figure 19:**
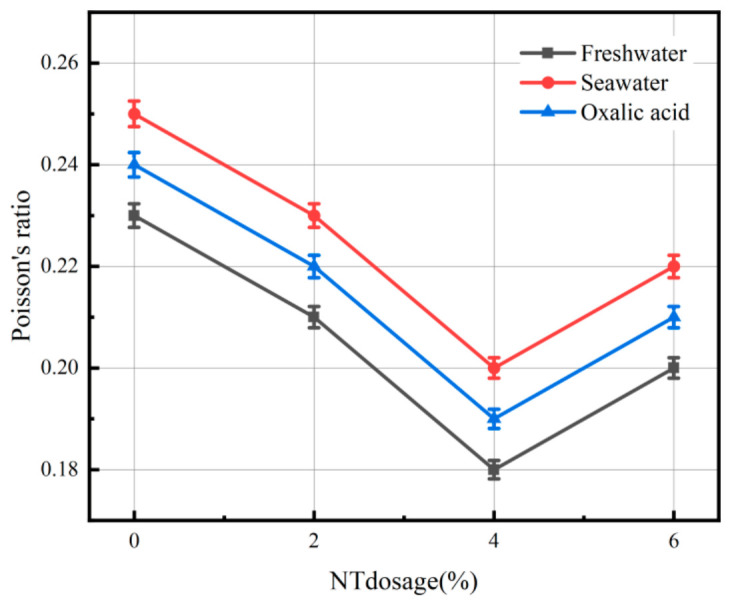
Relationship between Poisson’s ratio of modified coral concrete and nano-TiO_2_ dosage.

Figure 19 illustrates the relationship between the Poisson’s ratio of modified coral concrete and the dosage of nano-TiO_2_. As illustrated in the figure, the Poisson’s ratio of modified coral concrete in diverse water environments exhibits a pattern of initial decline and subsequent increase with rising nano-TiO_2_ dosage. The Poisson’s ratio of modified coral concrete attains its maximum value when the dosage reaches 4%. In freshwater environments, the Poisson’s ratio of modified coral concrete is observed to decrease by 8.70%, 21.74%, and 13.04% at nano-TiO_2_ contents of 2%, 4%, and 6%, respectively, in comparison to the ratio at a nano-TiO_2_ content of 0%. In seawater environments, the Poisson’s ratio of modified coral concrete is observed to decrease by 8.00%, 20.00%, and 12.00% at nano-TiO_2_ contents of 2%, 4%, and 6%, respectively, in comparison to the ratio at a nano-TiO_2_ content of 0%. The addition of nano-TiO_2_ at dosages of 2%, 4%, and 6% to an oxalic acid environment has been observed to result in a reduction in the concrete Poisson’s ratio by 8.33%, 20.83%, and 12.50%, respectively, in comparison to the nano-TiO_2_ dosage of 0%. The introduction of nano-TiO_2_ at a dosage of 6% has been found to result in an increase in the concrete Poisson’s ratio by 11.11%, 10.00%, and 10.53%, respectively, in comparison to the dosage of 4% in freshwater, seawater, and oxalic acid environments. This is primarily attributable to the observation that as the nano-TiO_2_ dosage increases, the compressive strength of the modified coral concrete demonstrates a gradual enhancement, which subsequently improves the axial compression deformation capacity of the coral concrete. However, the enhancement in the transverse deformation capacity of the coral concrete is less pronounced than that in the vertical deformation capacity. Consequently, the Poisson’s ratio of the test piece exhibits a slight decline with the increase in the dosage. When the dosage exceeds the optimal dosage, a slight increase in Poisson’s ratio of the test piece is observed. Nevertheless, the alteration in Poisson’s ratio remains minimal and essentially unchanging.

#### 3.7.2. Effect of Water Environment

**Figure 20:**
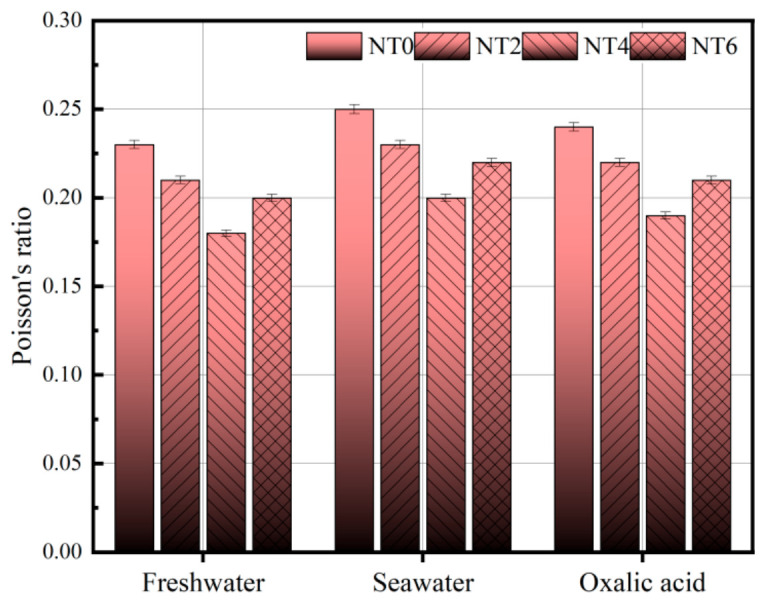
Relationship between Poisson’s ratio and the water environment of modified coral concrete.

Figure 20 illustrates the Poisson’s ratio of modified coral concrete in various aqueous environments. As illustrated in the figure, the Poisson’s ratio of modified coral concrete with an identical quantity of nano-titanium dioxide is the lowest in freshwater, followed by oxalic acid, and the highest in seawater. Furthermore, the mean Poisson ratios of modified coral concrete under the four nano-TiO_2_ dosages, in descending order, are as follows: the seawater group, the oxalic acid group, and the freshwater group. When the nano-TiO_2_ content is 0%, 2%, 4%, and 6%, the Poisson’s ratio of the modified coral concrete in the seawater environment is 8.70%, 9.52%, 11.11%, and 10.00% higher than that in the freshwater environment, respectively. Similarly, the Poisson’s ratio of the coral concrete in the oxalic acid environment is 4.35%, 4.76%, 5.56%, and 5.00% higher than that in the freshwater environment, respectively. This is primarily due to the differing reactions of seawater and oxalic acid solutions with the hydration products of cement, which in turn lead to alterations in the internal structure of concrete and changes in the properties of the transition zone at the interface between the cement and coral aggregates. These changes affect the transverse and vertical deformation capacity of the test piece.

### 3.8. Uniaxial Compression Test Parameter Relationship

#### 3.8.1. Relationship Between Elastic Modulus and Peak Stress

**Figure 21:**
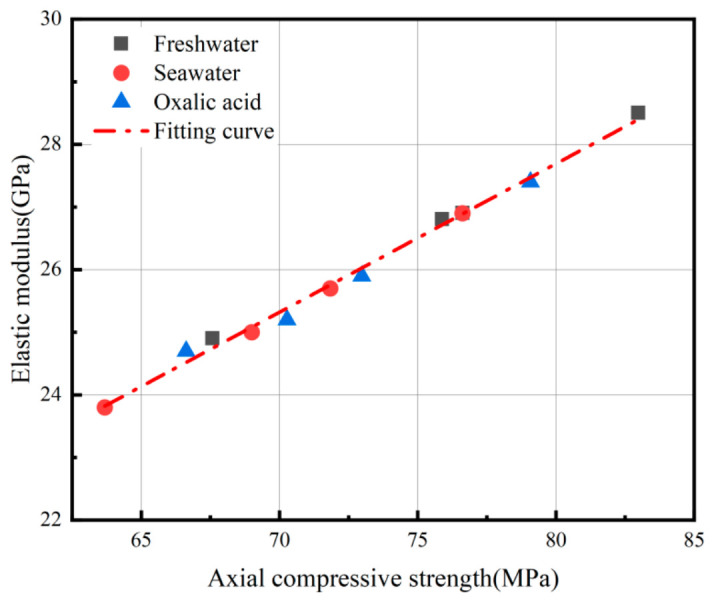
Fitting relationship between the elastic modulus and axial compressive strength of NTCC in different water environments.

Figure 21 illustrates the correlation between the elastic modulus and the axial compressive strength of NTCC. It is evident that there is a robust linear correlation between the elastic modulus and the axial compressive strength of NTCC. A linear regression analysis of the data yielded the following equation:

(1) $$E_{NTCC,c}=0.237f_{NTCC,cp}+8.723\;(R^{2}=0.993)$$

In the formula, $E_{NTCC,c}$ represents the elastic modulus of NTCC (GPa); $f_{NTCC,cp}$ represents the axial compressive strength of NTCC (MPa).

The correlation coefficient R^2^ of the fitted linear equation is 0.993, which indicates that the fitted linear equation is an effective means of calculating the elastic modulus and the axial compressive strength.

#### 3.8.2. Relationship Between Peak Strain and Peak Stress

Given that the concrete is in an elastic–plastic state prior to damage by axial compression, it is evident that generalized Hooke’s law is unable to accurately express the relationship in this state. Hooke’s law demonstrates that strain and stress have a first-order linear relationship. In order to characterize the curve of the relationship between the NTCC peak strain and peak stress, the relationship is fitted using the following equation:

(2) $$\varepsilon_{p}=a_{1}f_{cp}+b_{1}$$

In the formula, $\varepsilon_{p}$ represents peak strain (10^−3^) and $a_{1},b_{1}$ represents the correction factor.

**Figure 22:**
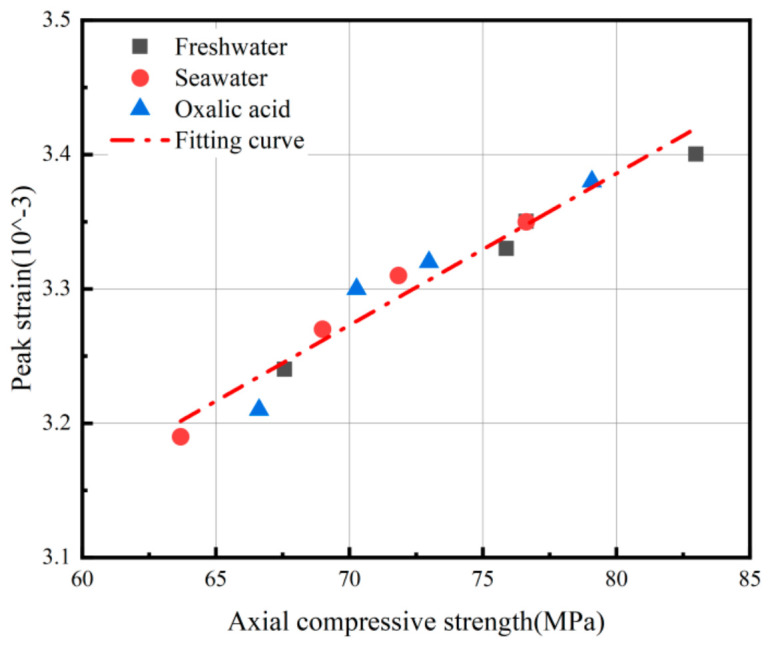
Fitting relationship between peak strain and axial compressive strength of NTCC in different water environments.

Figure 22 illustrates the correlation between peak strain and axial compressive strength of the NTCC, and the fitted relationship is expressed as follows:

(3) $$\varepsilon_{NTCC,p}=0.011f_{NTCC,cp}+2.482(R^{2}=0.950)$$

In the formula, $\varepsilon_{NTCC,p}$ represents peak strain of NTCC (10^−3^); $f_{NTCC,cp}$ represents axial compressive strength of NTCC (MPa).

The correlation coefficient R^2^ of the empirical formula is 0.950, and the comprehensive fitted curve relationship diagram demonstrates that the empirical formula can, to a reasonable extent, accurately characterize the relationship between the peak strain of NTCC and the axial compressive strength. However, when compared to the extent of deviation of the data points for freshwater and seawater from the fitted curve, the data points for the oxalic acid environment exhibit a greater degree of deviation from the fitted curve. As can be observed from Hooke’s law formula and Formula (2), the slope of the fitted curve is correlated with the reciprocal of the elastic modulus. It can be observed that the impact of the oxalic acid environment on the test piece may result in acid damage to the coral aggregate, which could subsequently lead to damage to the concrete skeleton structure. The effects of freshwater and seawater can be attributed primarily to the influence of the hydration products of the matrix. The impact of the two types of damage on the elastic modulus of the test piece exhibits a discernible discrepancy. Consequently, the deviation observed in the oxalic acid environment is more pronounced than that in freshwater and seawater.

### 3.9. Microstructural Pore Characteristics

**Figure 23:**
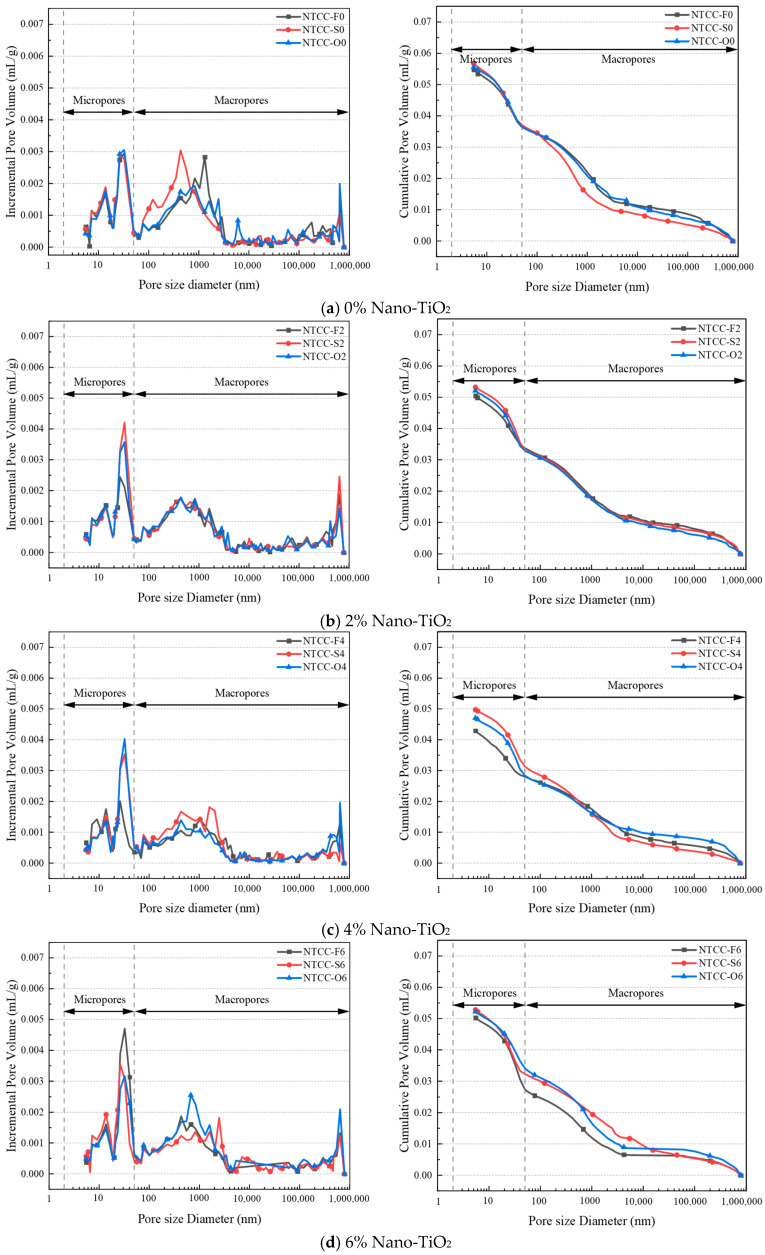
MIP results of NTCC with different nano-TiO_2_ dosages.

**Figure 24:**
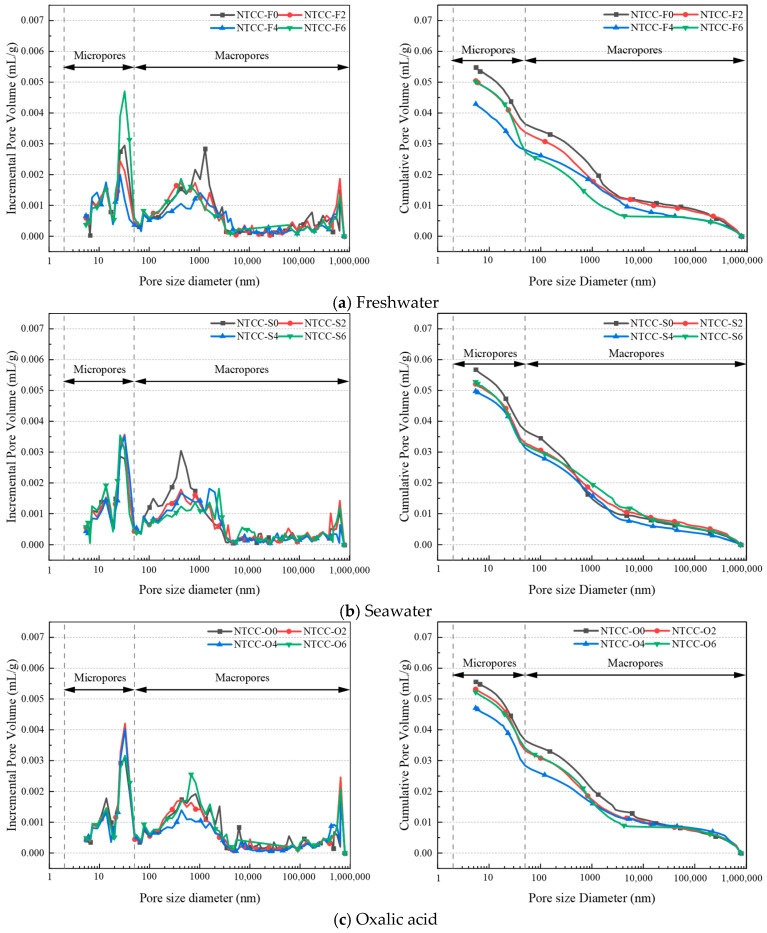
MIP results of NTCC with different curing environments.

The obtained MIP results are shown in Figure 23 and Figure 24, and the statistical pore parameters are listed in Table 4.

The MIP test results indicate that the total porosity of NTCC in all three curing environments first decreased and then increased with increasing nano-TiO_2_ dosage, reaching the minimum value at a dosage of 4%. When no nano-TiO_2_ was added (0%), the total porosity of NTCC cured in freshwater, seawater, and oxalic acid solution was 11.504%, 12.115%, and 11.883%, respectively. Compared with the control group (0% nano-TiO_2_), the total porosity in the freshwater environment decreased by 5.38%, 18.08%, and 8.10% at nano-TiO_2_ dosages of 2%, 4%, and 6%, respectively. In the seawater environment, the reductions were 6.85%, 10.94%, and 7.08%, respectively. In oxalic acid solution, the total porosity decreased by 8.33%, 20.83%, and 12.50% at the corresponding dosages. When the nano-TiO_2_ dosage increased from 4% to 6%, the total porosity increased by 12.18%, 4.33%, and 5.93% in freshwater, seawater, and oxalic acid environments, respectively. Additionally, the average pore diameter of NTCC in freshwater and oxalic acid environments exhibited a trend of first decreasing and then increasing with increasing nano-TiO_2_ dosage. In contrast, the average pore diameter in the seawater environment showed an opposite trend, first increasing and then decreasing.

According to the pore classification method [31], pores with diameters smaller than 20 nm are classified as harmless pores, pores between 20 and 50 nm as less harmful pores, and pores larger than 50 nm as harmful pores. The pore size distribution of NTCC specimens under the three curing environments was mainly concentrated in the ranges of 5–19 nm, 20–50 nm, 50–3755 nm, and 463,951–787,199 nm. Under the same curing environment, the proportions of harmless pores (<20 nm) and less harmful pores (20–50 nm) remained relatively consistent across different nano-TiO_2_ dosages. However, the proportion of harmful pores (>50 nm) first increased and then decreased with increasing nano-TiO_2_ content. Correspondingly, the cumulative porosity first decreased and then increased with increasing nano-TiO_2_ dosage. Overall, the pore structure of NTCC reached its optimal state at a nano-TiO_2_ dosage of 4% in all three water environments, where the total porosity was minimized. These results indicate that an appropriate amount of nano-TiO_2_ improves the pore structure of NTCC through its filling effect, which effectively reduces both the total porosity and the proportion of harmful pores, thereby enhancing the static and dynamic mechanical properties of the material. However, excessive nano-TiO_2_ tends to agglomerate within the concrete matrix, weakening the filling effect. This leads to an increase in total porosity and a deterioration of the static and dynamic mechanical performance.

For NTCC with the same nano-TiO_2_ dosage, the total porosity was lowest in freshwater, intermediate in oxalic acid solution, and highest in seawater. Specifically, compared with the freshwater environment, the total porosity in seawater increased by 5.31%, 3.67%, 14.49%, and 6.48% at nano-TiO_2_ dosages of 0%, 2%, 4%, and 6%, respectively. In the oxalic acid environment, the total porosity increased by 3.29%, 3.53%, 9.54%, and 3.43% relative to freshwater at the corresponding dosages. At a nano-TiO_2_ dosage of 4%, the average pore diameter of NTCC was smallest in freshwater, followed by the oxalic acid environment, and largest in seawater. When the nano-TiO_2_ dosage was 4% or lower, the proportions of harmless pores (<20 nm) and less harmful pores (20–50 nm) were lowest in freshwater, intermediate in oxalic acid solution, and highest in seawater under the same dosage. At 6% nano-TiO_2_ dosage, there was no significant difference in the proportion of harmless pores among the three environments, while the proportion of less harmful pores was lowest in oxalic acid, intermediate in seawater, and highest in freshwater. However, the proportion of harmful pores (>50 nm) was consistently lowest in freshwater, followed by oxalic acid solution, and highest in seawater under the same nano-TiO_2_ dosage. Correspondingly, the cumulative porosity of NTCC specimens followed the same order: lowest in freshwater, intermediate in oxalic acid solution, and highest in seawater at the same nano-TiO_2_ dosage.

## 4. Constitutive Model

### 4.1. Establishment of the Constitutive Models

The existing research demonstrates that the full stress–strain curve of coral concrete comprises an ascending and a descending section. As illustrated in Figure 21 and Figure 22, the full stress–strain curve of coral concrete should exhibit the following characteristics:

(1) The curve in question passes through the origin when x=0, y=0;

(2) The segment in question displays an outward bulging curvature when $0\leq x\leq 1,\frac{d^{2}y}{dx^{2}}\leq 0$.

(3) The slope of the tangent line to the curve at the peak point is equal to zero when $x=1,y=1,\frac{dy}{dx}=0$.

(4) The curve exhibits a turning point in its downward section and a point of greatest curvature.

(5) The curve in question possesses a horizontal asymptote on the x-axis when $x\to \infty ,y\to 0,\frac{dy}{dx}\to 0$.

(6) The curve coordinates satisfy the condition x≥0,0≤y≤1.

For the uniaxial compression stress–strain full curve equation of concrete, scholars at home and abroad have proposed a variety of mathematical models to describe its equation, including polynomial model, exponential model, trigonometric function-based model and rational fractional model. According to China’s current national standard GB 50010-2010 [32], the complete stress–strain curve equation for concrete under uniaxial compression proposed by Guo et al. [33] was adopted in this study to describe the mechanical behavior of coral concrete. The expression is as follows:

(4) $$y(x)=\left\{\begin{array}{ll} ax+\left(3-2a\right)x^{2}+\left(a-2\right)x^{3} & 0\leq x\leq 1\\ \frac{x}{b{\left(x-1\right)}^{2}+x} & x>1 \end{array}\right.$$

In the formula $x=\varepsilon /\varepsilon_{p}$, ε represents strains, $\varepsilon_{p}$ represents peak strain; in $y=\sigma /\sigma_{p}$, σ represents stress, $\sigma_{p}$ represents peak stress, a represents curve control parameter for Rise Phase, and b represents curve control parameter for Descent Phase.

In a comprehensive analysis, Equation (4) is more accurate in fitting the rising section of the curve, although there is a significant discrepancy in the falling section. Consequently, this paper proposes a correction based on Equation (4), which yields the following NTCC stress–strain full curve equation:

(1) Rising segment of the curve:

(5) $$y=ax+(3-2a)x^{2}+(a-2)x^{3}$$

(2) Downward sloping section of the curve:

(6) $$y=\frac{x}{b{\left(x-1\right)}^{c}+x}$$

In the formula, a represents the curve control parameter for Rise Phase, b and c represent the curve control parameters for Descent Phase. The curve-fitting parameters for each group are presented in Table 5.

### 4.2. Verification of the Established Constitutive Models

**Figure 25:**
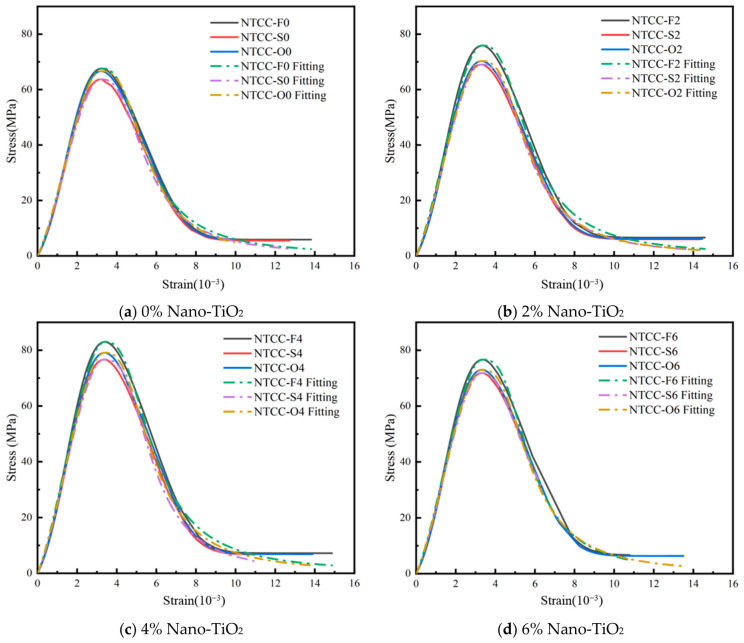
Comparison of the uniaxial compressive stress–strain test full curve and fitted full curve for NTCC.

As illustrated in Table 5, the correlation coefficients of the fitted parameters for both the ascending and descending segments of the stress–strain full curve exceed 0.98. Figure 25 illustrates the comparison between the NTCC uniaxial compressive stress–strain test curve and the fitted curve. It can be observed that the test line aligns closely with the fitted curve, indicating a high degree of correlation. The synthesis of Figure 25 and Table 5 demonstrates that Equations (5) and (6) are more effective at expressing the NTCC uniaxial compressive stress–strain full curve equations.

## 5. Conclusions

This study examines the mechanical properties and uniaxial compressive stress–strain relationship of NTCC with varying dosages of TiO_2_ nanoparticles. Its performance is analyzed and evaluated when applied to diverse aqueous environments, including freshwater, seawater, and oxalic acid solution conservation. Additionally, an empirical segmented ontological model is proposed. In light of the test results and ensuing discussions, the following conclusions may be drawn.

(1) The doping of nano-TiO_2_ has a positive effect on the basic mechanical properties of NTCC, whereas an excessive amount of nano-TiO_2_ has a detrimental impact on these properties. The results demonstrated that when the doping of nano-TiO_2_ was within 6%, the cubic compressive strength, split tensile strength, axial compressive strength and modulus of elasticity of NTCC exhibited a trend of increasing and then decreasing with the increase in doping. The optimal dosage was identified as 4%, at which point the various properties reached their maximum value. The cubic compressive strength, split tensile strength, axial compressive strength and modulus of elasticity of NTCC with the optimal dosage exhibited an increase of approximately 22%, 20%, 22% and 14%, respectively, compared with those with zero dosage.

(2) The seawater environment contributes to the early compressive strength of NTCC, with a 7-day compressive strength that can reach 90% of the 28-day compressive strength. However, both seawater and oxalic acid environments have an adverse effect on the late 28-day strength. The values of the cubic compressive strength, split tensile strength, axial compressive strength and modulus of elasticity of NTCC at 28 days in different water environments demonstrated that the seawater environment exhibited the lowest values, while the oxalic acid environment exhibited the second lowest values, and the freshwater environment exhibited the highest values.

(3) The compressive damage of NTCC exposed to different water environments and with varying levels of TiO_2_ dosages was found to exhibit a greater propensity towards brittle failure. In the absence of nano-TiO_2_, NTCC exhibited significant concrete spalling in diverse water environments. However, with the augmentation of nano-TiO_2_ dosage, the damage degree exhibited a gradual decline. Furthermore, the freshwater group exhibited the lowest degree of damage among the three groups.

(4) The doping of nano-TiO_2_ enhanced the quasi-static mechanical compression performance of NTCC. The nano-TiO_2_ dosage of 4% proved optimal, while both seawater and oxalic acid exerted a deleterious effect on the quasi-static mechanical properties of NTCC. The uniaxial compressive stress–strain curves of NTCC under different water environments and different dosages of nano-TiO_2_ can be divided into three distinct stages: an elastic stage, an elastic–plastic stage, and a decreasing stage.

(5) The upward and downward sections of the uniaxial compressive stress–strain curves were fitted by the modified constitutive equation provided in the specification, and the correlation coefficients of the fitted parameters in the aforementioned sections were found to be above 0.98. The fitted curves demonstrated a high degree of correlation with the experimental curves, thereby substantiating the efficacy of the modified stress–strain equations in accurately representing the uniaxial compressive stress–strain curves of NTCC under diverse aqueous environments and varying nano-TiO_2_ dosages.

## Figures and Tables

**Table 1:** Chemical compositions of Portland cement, fly ash and slag (%).

Type	CaO	SiO_2_	Al_2_O_3_	MgO	SO_3_	Fe_2_O_3_	K_2_O	Na_2_O
Portland cement	62.53	22.59	5.27	3.66	2.36	3.52	0.51	0.43
fly ash	4.92	45.75	29.07	1.56	0.75	6.09	0.90	0.59
slag	43.26	30.62	14.07	6.09	2.36	0.71	0.46	0.396

**Table 2:** Artificial seawater mixture ratio (g/L).

NaCl	MgCl_2_	Na_2_SO_4_	CaCl_2_	KCl	NaHCO_3_	H_2_O
22.16	5.265	3.861	01.082	0.745	0.207	1000

**Table 3:** Mix proportion of nano-modified coral concrete (kg/m^3^).

No.	Cement	Slag	Fly Ash	Nano-TiO_2_	Coral Coarse Aggregate	Coral Sand	Water			Water-Reducing Agent
							Freshwater	Seawater	Oxalic Acid Solution	
NTCC-F0	780	150	70	0	300	700	250	-	-	20
NTCC-F2	760	150	70	20	300	700	250	-	-	20
NTCC-F4	740	150	70	40	300	700	250	-	-	20
NTCC-F6	720	150	70	60	300	700	250	-	-	20
NTCC-S0	780	150	70	0	300	700	-	250	-	20
NTCC-S2	760	150	70	20	300	700	-	250	-	20
NTCC-S4	740	150	70	40	300	700	-	250	-	20
NTCC-S6	720	150	70	60	300	700	-	250	-	20
NTCC-O0	780	150	70	0	300	700	-	-	250	20
NTCC-O2	760	150	70	20	300	700	-	-	250	20
NTCC-O4	740	150	70	40	300	700	-	-	250	20
NTCC-O6	720	150	70	60	300	700	-	-	250	20

**Table 4:** MIP results of NTCC at 28 days.

Serial Number	Porosity (%)	Pore Size Distribution (%)			Average Pore Diameter (nm)	Median Pore Diameter (nm)
		<20 nm	20–50 nm	>50 nm		
NTCC-F0	11.504	15.286	18.340	66.374	46.77	14.39
NTCC-F2	10.885	16.355	16.846	66.798	44.54	13.54
NTCC-F4	9.424	20.583	14.040	65.378	38.58	11.18
NTCC-F6	10.572	15.685	29.751	54.564	39.76	17.91
NTCC-S0	12.115	16.569	18.213	65.218	43.78	15.08
NTCC-S2	11.285	15.214	21.530	63.256	44.6	15.96
NTCC-S4	10.790	14.290	22.801	62.909	45.74	16.86
NTCC-S6	11.257	17.757	21.137	61.106	40.82	15.17
NTCC-O0	11.883	14.716	19.377	65.907	48.67	16.39
NTCC-O2	11.269	14.035	23.498	62.467	46.08	16.99
NTCC-O4	10.323	15.014	24.707	60.278	42.94	15.98
NTCC-O6	10.935	14.489	20.262	65.249	46.43	15.39

**Table 5:** Fitting parameters of the NTCC stress–strain full curve equation.

Serial Number	a	$R_{Rise}^{2}$	b	c	$R_{Descent}^{2}$
NTCC-F0	1.66	0.987	3.91	2.84	0.993
NTCC-F2	1.72	0.985	3.70	2.90	0.993
NTCC-F4	1.78	0.993	3.78	2.87	0.993
NTCC-F6	1.74	0.994	4.12	3.06	0.998
NTCC-S0	1.63	0.998	3.80	2.93	0.989
NTCC-S2	1.68	0.997	3.71	2.99	0.993
NTCC-S4	1.75	0.998	4.16	3.13	0.997
NTCC-S6	1.70	0.989	3.08	2.62	0.998
NTCC-O0	1.65	0.987	3.98	3.10	0.997
NTCC-O2	1.69	0.996	3.85	2.97	0.994
NTCC-O4	1.76	0.985	3.98	2.94	0.992
NTCC-O6	1.71	0.989	3.79	2.97	0.994

## Data Availability

The original contributions presented in this study are included in the article. Further inquiries can be directed to the corresponding author.

## References

[1] Qin Q., Wu K., Meng Q., Gan M., Yi P. (2024). Investigation of mechanical characterization and damage evolution of coral reef sand concrete using in-situ CT and digital volume correlation techniques. J. Build. Eng..

[2] de Sousa M.F., da Natividade C.D., Filho M.R.F.L., Torres S.M., dos Santos A.J.V., da Rocha R.A.S., Henriques G.F., Massei K., de Souza W.M. (2025). Mineralogical and Mechanical Characterization of Concrete Blocks for Artificial Reefs: A Comparative Study with Natural Coral Skeletons. J. Mar. Sci. Eng..

[3] Zhang B., Yang Z., Jiang X., Zhu H., Wang W., Dong Y.-R., Peng H. (2024). Flexural behavior of coral aggregate concrete beams re-inforced with BFRP bars under seawater corrosion environments. Constr. Build. Mater..

[4] Linaraki D. (2025). Design and Fabrication of Bio-Enhancing Surfaces for Coral Settlement. Architecture.

[5] Chao Z., Li Z., Dong Y., Shi D., Zheng J. (2024). Estimating compressive strength of coral sand aggregate concrete in marine environment by combining physical experiments and machine learning-based techniques. Ocean Eng..

[6] Ma L., Li Z., Liu J., Duan L., Wu J. (2019). Mechanical properties of coral concrete subjected to uniaxial dynamic compression. Constr. Build. Mater..

[7] Huang Y., He X., Sun H., Sun Y., Wang Q. (2018). Effects of coral, recycled and natural coarse aggregates on the mechanical properties of concrete. Constr. Build. Mater..

[8] Wang G., Wu Q., Zhou H., Peng C., Chen W. (2021). Diffusion of chloride ion in coral aggregate seawater concrete under marine environment. Constr. Build. Mater..

[9] Zhou W., Feng P., Lin H. (2020). Constitutive relations of coral aggregate concrete under uniaxial and triaxial compression. Constr. Build. Mater..

[10] Tan Y., Yu H., Mi R., Zhang Y. (2018). Compressive strength evaluation of coral aggregate seawater concrete (CAC) by non-destructive techniques. Eng. Struct..

[11] Cai Y., Ren H.-Q., Long Z.-L., Guo R.-Q., Du K.-M., Chen S.-S., Zheng Z.-H. (2022). Comparison study on the impact compression mechanical proper-ties of coral aggregate concrete and ordinary Portland concrete. Structures.

[12] Huang D., Niu D., Su L., Fu Q. (2021). Chloride diffusion behavior of coral aggregate concrete under drying-wetting cycles. Constr. Build. Mater..

[13] Cao Y., Bao J., Zhang P., Sun Y., Cui Y. (2022). A state-of-the-art review on the durability of seawater coral aggregate concrete exposed to marine environment. J. Build. Eng..

[14] Bayraktar O.Y., Danish A., Bodur B., Kaplan G., Aydın A.C., Ozbakkaloglu T. (2024). Performance assessment of fiber-reinforced coral aggregate-based lightweight foam concrete for sustainable marine construction. Constr. Build. Mater..

[15] Huang D., Niu D., Su L., Pan D., Liu Y. (2022). Durability of coral aggregate concrete under coupling action of sulfate, chloride and drying-wetting cycles. Case Stud. Constr. Mater..

[16] Zhang B., Zhu H., Chen J. (2023). Bond durability between BFRP bars and seawater coral aggregate concrete under seawater corro-sion environments. Constr. Build. Mater..

[17] Guo J., Zhang J., Yu H., Ma H. (2023). Dynamic compressive behaviour of basic magnesium sulfate cement–coral aggregate concrete (BMSC–CAC) after exposure to elevated temperatures: Experimental and analytical studies. Constr. Build. Mater..

[18] Guo J., Yu H., Ma H., Wu Z. (2022). Damage and deterioration characteristics of basic magnesium sulfate cement-coral aggregate concrete exposed to elevated temperature. Eng. Fail. Anal..

[19] Zhang L., Niu D., Wen B., Fu Q., Zhang Y. (2021). Corrosion rate models of reinforcement in modified coral aggregate concrete. Constr. Build. Mater..

[20] Zhang W., Xie X., Shi D., Shao W., Zhang J. (2025). Experimental insights into the mechanical properties and constitutive models of coral aggregate seawater concrete mixed with natural aggregates. J. Build. Eng..

[21] Guo J., Zhang J., Yu H., Ma H., Wu Z. (2022). Experimental and 3D mesoscopic investigation of uniaxial compression performance on basic magnesium sulfate cement-coral aggregate concrete (BMSC-CAC). Compos. Part B Eng..

[22] Zhang B., Zhu H. (2023). Compressive stress–strain behavior of slag-based alkali-activated seawater coral aggregate concrete after exposure to seawater environments. Constr. Build. Mater..

[23] Tanimola J.O., Efe S. (2024). Recent advances in nano-modified concrete: Enhancing durability, strength, and sustainability through nano silica (nS) and nano titanium (nT) incorporation. Appl. Eng. Sci..

[24] Zhou Y., Zhuang J., Xu W., Lin W., Xing F., Hu R. (2024). Study on mechanical performance and mesoscopic simulation of nano-SiO_2_ modified recycled aggregate concrete. Constr. Build. Mater..

[25] Fan X.-C., Zhang A., Gao X., Qin Y. (2023). Effect of water environment on mechanical behavior of coral aggregate concrete. Mar. Georesources Geotechnol..

[26] Bunyamin B., Kurniasari F.D., Munirwan R.P., Jaya R.P. (2022). Effect of Coral Aggregates of Blended Cement Concrete Sub-jected to Different Water Immersion Condition. Adv. Civ. Eng..

[27] Chen M., Geng J., Xiong H., Shang T., Xue C., Abbas M. (2020). Effect of Curing on Mechanical Properties of Cement-Stabilized Cor-al Sand in Marine Environment. Adv. Mater. Sci. Eng..

[28] Da B., Li Y., Yu H., Ma H., Chen H., Dou X., Wu Z. (2022). Effect of Carbonation and Drying-Wetting Cycles on Chloride Diffusion Behavior of Coral Aggregate Seawater Concrete. J. Ocean Univ. China.

[29] Qin Y., Wang Q., Xu D., Chen W. (2021). Mechanical Behavior and Healing Efficiency of Microcapsule-Based Cemented Coral Sand under Various Water Environments. Materials.

[30] (2019). Standard for Test Methods of Physical and Mechanical Properties of Concrete.

[31] Houben M., Desbois G., Urai J. (2014). A comparative study of representative 2D microstructures in Shaly and Sandy fa-cies of Opalinus Clay (Mont Terri, Switzerland) inferred form BIB-SEM and MIP methods. Mar. Pet. Geol..

[32] (2014). Code for Design of Concrete Struc-Tures.

[33] Guo Z. (2014). Principles of Reinforced Concrete.

